# Odin (ANKS1A) is a Src family kinase target in colorectal cancer cells

**DOI:** 10.1186/1478-811X-6-7

**Published:** 2008-10-09

**Authors:** Muhammad Emaduddin, Mariola J Edelmann, Benedikt M Kessler, Stephan M Feller

**Affiliations:** 1 Cell Signalling Group, Department of Molecular Oncology, Weatherall Institute of Molecular Medicine, John Radcliffe Hospital, Oxford University, Headley Way, Oxford OX3 9DS, UK; 2 Central Proteomics Facility (CPF), Henry Wellcome Building for Molecular Physiology, Department of Clinical Medicine and Weatherall Institute of Molecular Medicine, John Radcliffe Hospital, Oxford University, Headley Way, Oxford OX39DS, UK

## Abstract

**Background:**

Src family kinases (SFK) are implicated in the development of some colorectal cancers (CRC). One SFK member, Lck, is not detectable in normal colonic epithelium, but becomes aberrantly expressed in a subset of CRCs. Although SFK have been extensively studied in fibroblasts and different types of immune cells, their physical and functional targets in many epithelial cancers remain poorly characterised.

**Results:**

64 CRC cell lines were tested for expression of Lck. SW620 CRC cells, which express high levels of Lck and also contain high basal levels of tyrosine phosphorylated (pY) proteins, were then analysed to identify novel SFK targets. Since SH2 domains of SFK are known to often bind substrates after phosphorylation by the kinase domain, the LckSH2 was compared with 14 other SH2s for suitability as affinity chromatography reagent. Mass spectrometric analyses of LckSH2-purified pY proteins subsequently identified several proteins readily known as SFK kinase substrates, including cortactin, Tom1L1 (SRCASM), GIT1, vimentin and AFAP1L2 (XB130). Additional proteins previously reported as substrates of other tyrosine kinase were also detected, including the EGF and PDGF receptor target Odin. Odin was further analysed and found to contain substantially less pY upon inhibition of SFK activity in SW620 cells, indicating that it is a formerly unknown SFK target in CRC cells.

**Conclusion:**

Rapid identification of known and novel SFK targets in CRC cells is feasible with SH2 domain affinity chromatography. The elucidation of new SFK targets like Odin in epithelial cancer cells is expected to lead to novel insight into cancer cell signalling mechanisms and may also serve to indicate new biomarkers for monitoring tumor cell responses to drug treatments.

## Background

### Src family kinases (SFK) in human cancers

SFK play crucial roles in a wide range of human signalling pathways and cell types. They are also implicated in several human cancer types, including colorectal cancers [1]. For historical reasons, many studies looking at SFK signalling and SFK-driven oncogenesis were initially done with avian and mammalian fibroblasts and later on in a variety of haematopoietic cells [2]. Much less is known about the actions and targets of SFK in epithelial cells, which account for the majority of human tumors. c-Src and other SFK members appear to be rarely mutated in human tumors, a fact that has led to their delayed recognition as therapeutic targets for cancer treatments [3]. Further complexity arises from the great heterogeneity of molecular lesions found in human tumors [4], which is only now becoming fully appreciated.

A recent study from our group with a large panel of human CRC cell lines has shown that most, if not all CRC cells require a basal SFK activity for proliferation and also identified c-Met as a target of SFK in a subset of CRC cells with highly active SFK [5]. Many other substrates of SFK remain unknown. Further roles of SFK in CRC cell migration, invasion etc. have been described but are only partially understood with respect to the molecular events that occur (reviewed in [1]).

Nevertheless, inhibitors with SFK blocking activity are currently making their way into the clinic, for example as second generation tyrosine kinase inhibitors for CML therapy. In addition, several SFK inhibitor trials for solid tumors like colorectal carcinomas are ongoing or in the planning phase [6]. A better understanding of the roles and effectors of SFK in CRC cells is therefore urgently needed.

In order to learn more about SFK targets in CRC, we have initiated a mass spectrometry based analysis of tyrosine phosphorylated (pY) proteins using a panel of CRC cells. In this pilot study we focus on SW620 cells which aberrantly express Lck and test the usefulness of SH2 domains as affinity purification reagents. Our experiments show that this is a viable technique to rapidly identify novel SFK targets, which should also be applicable to many other signalling systems that depend on tyrosine phosphorylation of proteins.

## Results

### Expression of Lck in a subset of CRC cells

**Figure 1:**
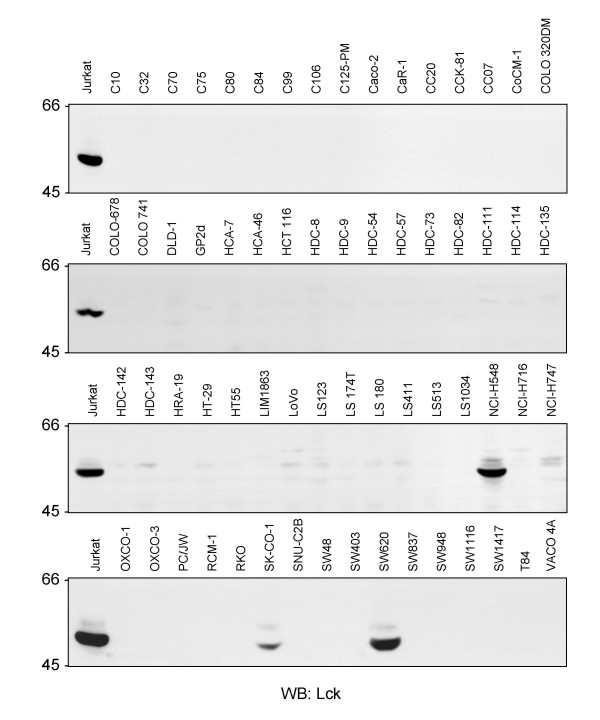
**Aberrant expression of the Src family kinase Lck in three of sixty-four CRC cell lines**. 150 μg of total cell RIPA lysates (TCL) from each cell line were separated by SDS-PAGE and analysed for Lck expression by western blotting. 150 μg of TCL from the Jurkat T-cell line were also used as positive control.

**Figure 2:**
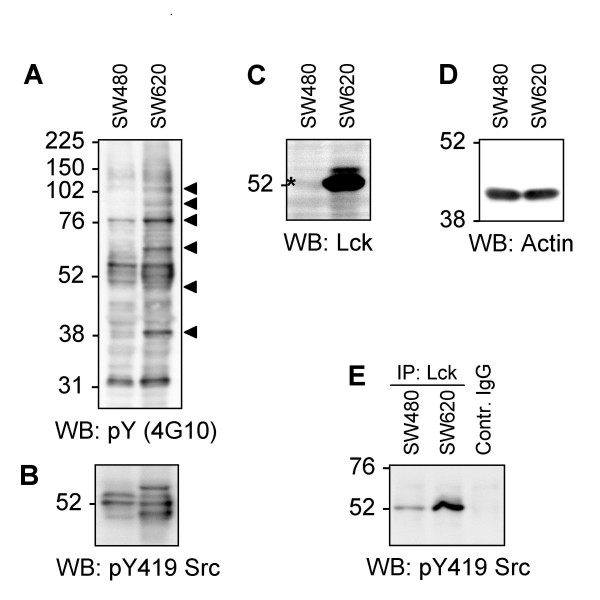
**Comparison of SW480 and SW620 cells for overall pY levels, SFK activity, Lck expression and activity**. Both cell lines are derived from the same patient. SW480 was established from the primary tumor, SW620 from a lymph node metastasis. A – D: 20 μg of TCL from each cell line were separated by SDS-PAGE and western blots (WB) conducted with antibodies as indicated. Arrowheads in A indicate pY bands increased or only detectable in SW620 cells. The asterisk in C indicates a barely visible band that comigrates with the expected size of Lck. Actin levels were analysed as loading control in D. To determine the phosphorylation of Lck in the activation loop (results shown in E), which critically regulates Lck activity, 1 mg of TCL from each cell line was immunoprecipitated with anti-Lck mAb and probed with anti-pY419Src that cross-reacts with several SFK family members, including Lck, due to high conservation of this epitope (further details in [5]). An IgG control of the same isotype was also included to precipitate non-specific binding proteins from 1 mg of TCL of SW620 cells. Note that some of the molecular weight markers used do not run exactly accordingly to the indicated molecular weights of the marker proteins, presumably due to the coupling of dye molecules to them.

A panel of 64 CRC cell lines (for further details on origins see [5], supporting information Table 1) was analysed for the expression of Lck by western blotting. Three lines with substantial expression of Lck protein, namely NCI-H548, SKCO-1 and SW620 cells, were detected (Figure 1). The SW620 cell line, which is derived from a tumor lymph node metastasis and contains particularly high levels of pY proteins [5], was selected for further analyses. It is noteworthy, that c-Src is not highly upregulated or activated in SW620 cells (see [5], supporting information Fig.12 for details). The protein expression and kinase activity status of other SFK has not yet been analysed comprehensively in these cells. To determine whether Lck is not only upregulated in its expression but also activated in SW620, these cells as well as SW480 cells, another cell line derived from the same patients' primary tumor, were compared for cell morphology (Additional file 1), Lck protein expression, total phosphotyrosine levels, overall activation of SFK and Lck activity (Figure 2). Interestingly, Lck protein expression is substantially higher in the metastasis-derived SW620 cells. This is also echoed in the Lck kinase activity, as indicated by Lck activation loop phosphorylation, as well as overall SFK activity and total tyrosine phosphorylation levels. However, whether Lck upregulation and activation is a key event in driving the development of tumor metastasis in some CRC remains to be investigated.

### Effective binding of phosphotyrosyl proteins from SW620 to the LckSH2 domain

**Figure 3:**
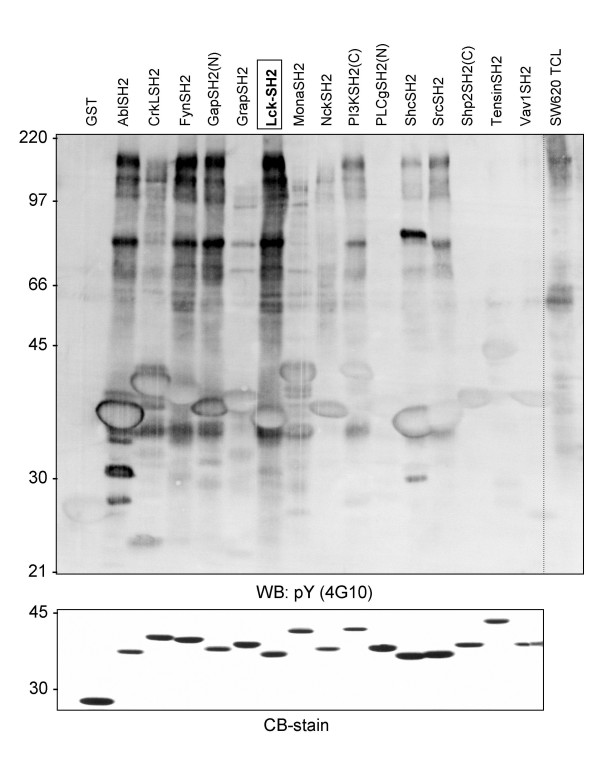
**Differential binding of pY proteins from SW620 cells to SH2 domains from 15 different signalling proteins**. Top panel: 1 mg of TCL was precipitated with 50 μg of bead-immobilised GST-SH2 fusion protein, precipitates repeatedly washed with RIPA buffer and bound proteins analysed by anti-pY western blotting. Bottom panel: Coomassie blue staining of the affinity purified GST-SH2 domain fusion proteins used for the precipitations. Approximately 10 μg of each protein was loaded onto the gel to analyse protein purity and integrity.

**Figure 4:**
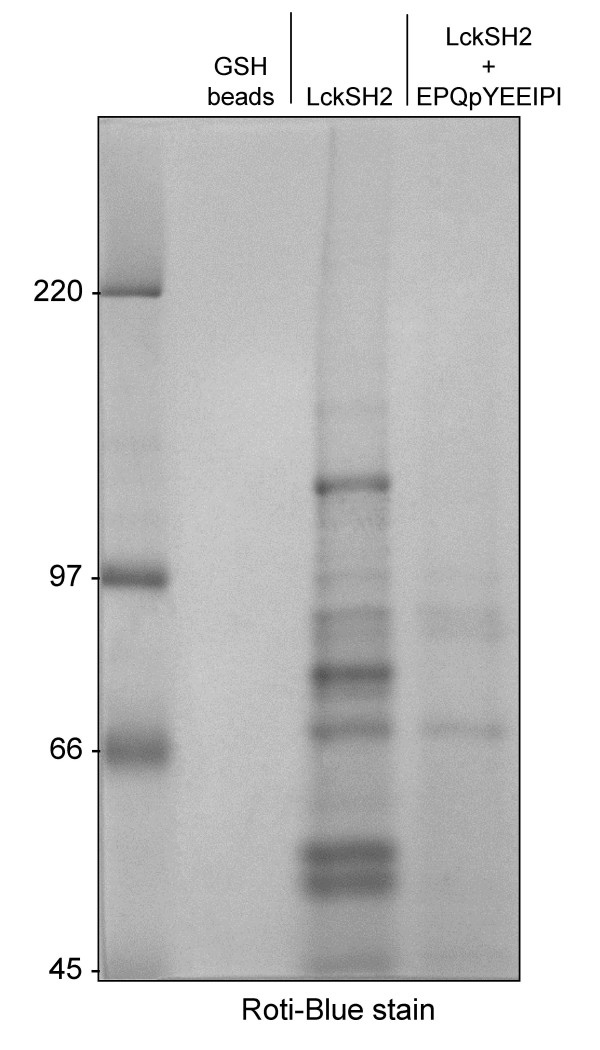
**Comparison of SW620 cytosolic proteins binding to LckSH2 versus LckSH2 pre-blocked with a high affinity binding pY peptide**. Cytosol (S100) of SW620 cells was pre-cleared several times with GST immobilised on GSH beads to reduce non-specific protein binding. Supernatants of these pre-clearings corresponding to 20 mg of S100 were then incubated with bead-immobilised GST-LckSH2 or GST-LckSH2 pre-blocked with the peptide EPQpYEEIPI, or unloaded GSH beads to detect potentially remaining non-specific protein binding and, after washing repeatedly with RIPA buffer, separated by SDS-PAGE and stained with colloidal dye (Roti-Blue). The GST-LckSH2 precipitation lane was subsequently processed for MS identification of bound proteins.

Src family kinases have been reported to initially phosphorylate substrates via their catalytic domain and to subsequently bind to the newly generated phosphoepitope via their SH2 domains, leading, at least in some cases, to processive phosphorylation of multiple tyrosines [7]. SFK SH2 domains could therefore be particularly useful tools to effectively affinity-purify pY proteins that are substrates of SFK. This assumption was tested subsequently with total cell SW620 lysate. 15 SH2 domains derived from different signalling proteins were expressed as GST-fusion proteins and used to precipitate pY proteins from SW620. Western blotting with anti-pY antibody showed that the LckSH2 precipitates a large number of pY proteins (Figure 3). Most other SH2 domains tested were less effective and the LckSH2 domain was chosen for subsequent pY protein purifications. Since pY proteins comprise only a very small fraction of cellular proteins, an additional cell fractionation step was combined with SH2 domain affinity purification step to isolate proteins for mass spectrometric (MS) analyses. Comparison of pY protein levels in different membrane fractions and cytosolic protein (S100) indicated that the vast majority of detectable pY proteins is found in the S100 (data not shown) and this fraction was further investigated. Several batches of 15 to 60 mg S100 protein were repeatedly pre-cleared with GST coupled to GSH-sepharose beads and finally GSH-beads alone to remove non-specific binding proteins and the remaining supernatant was then incubated with GST-LckSH2 beads. As a control, an equal amount of pre-cleared S100 was incubated with bead-immobilised GST-LckSH2, which had been pre-incubated with an excess of a phosphopeptide (EPQpYEEIPI; originally derived from hamster middle T antigen) that binds with high affinity to the pY binding pocket of the LckSH2 domain [8,9], or GSH beads alone. Bound proteins were detected with Coomassie Blue staining. A representative example is depicted in Figure 4. These experiments showed that most of the SW620 S100 proteins that interact with the LckSH2 require indeed the pY binding pocket of this domain. The gel lanes containing the proteins precipitated with GST-LckSH2 in the absence of pY peptide were subsequently cut into 10 slices, which were subjected to tryptic digest and MS analyses. The proteins identified through this approach are listed in Additional file 2.

### LckSH2 affinity chromatography allows rapid mass spectrometric identification of numerous tyrosine kinase substrates

Notably, many of the proteins identified are readily known as substrates of protein tyrosine kinases. Previously detected phosphosites for the MS-identified proteins were extracted from the PhosphoSitePlus™ database  and are also listed in Additional file 2. As this database is constantly updated and several other phosphoprotein databases exist, this listing is expected to underestimate the number of actual pY sites and pY proteins that were previously reported. Cortactin, a prominent SFK substrate [10], appeared as a major hit in several gel slices, which could be a consequence of differential splicing, the presence of posttranslational modifications or proteolytic processing in SW620 cells. The AceView program  predicts numerous putative isoforms for cortactin, but the splice-variants occurring in colonic epithelium or colorectal cancers have not yet been reported. Other known SFK substrates identified were vimentin [11,12], 3BP-2 (SH3BP2) [13], GIT1 [14], Tom1L1 [15] and AFAP1L2/XB130 [16]. The adaptor protein CRKL, the major target of the Bcr-Abl oncogene [17], a tyrosine kinase activating SFK in CML cells, was also detected. Other proteins found by MS, for example MAP1B, have only a marginal publication history of tyrosine phosphorylation, but contain multiple pY residues according to a publicly accessible databases . Yet others, like sorbitol dehydrogenase, are not yet established as tyrosine kinase targets but have been reported to regulate tyrosine kinase signalling [18]. Somewhat surprisingly, we did not find in our experiments some well-studied Src substrates like focal adhesion kinase (FAK), p130Cas/BCAR1 or p70 paxillin, which were initially reported as SFK substrates in fibroblasts. If this is due to technical limitations or whether these proteins are actually not SFK targets in SW620 cells remains to be determined.

Some examples of frequent 'false positives' found in many MS experiments (see also [19] for discussion) are underlined in the table. However, it should be noted that many of these proteins, for example tubulins and β-actin, have actually been reported to be tyrosine phosphorylated by SFK [20,21], so it is by no means certain or even likely that all of these proteins are non-specifically interacting with the SH2 domain affinity matrix.

### Odin is a target of SFK in CRC cells

From the marginally characterised pY proteins found by mass spectrometry that are not known SFK substrates, the Odin protein was selected for further experimental investigation. Odin has been first described as a target and signal transmitter of receptor tyrosine kinases like EGFR and PDGFR [22] and more recently as an interaction partner of the receptor tyrosine kinase EphA8 [23]. Odin reduction by siRNA diminishes ephrinA5-induced effects like cell migration in HEK293 cells and neurite retraction in Neuro2a cells.

**Figure 5:**
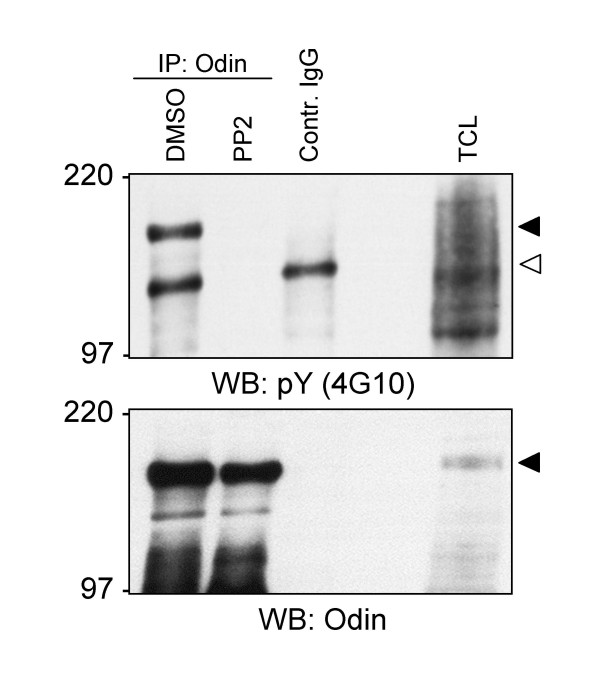
**Treatment of SW620 cells with the SFK inhibitor PP2 abolishes the tyrosine phosphorylation of Odin**. SW620 cells were treated with 30 μM of the SFK inhibitor PP2 dissolved in DMSO or 0.06%(v/v) DMSO for 24 h, then lysed and subjected to immunoprecipitation with anti-Odin or normal rabbit IgG (contr. IgG) as indicated. pY protein was detected by western blotting with anti-pY mAb. 10 μg of untreated SW620 TCL were also analysed. The blot was subsequently stripped and reprobed to detect Odin. The black arrowheads indicate the Odin bands, the open arrowhead points to a protein that binds non-specifically to control IgG beads and is equally affected by PP2 treatment of the SW620 cells.

As expected, Odin was precipitated with the Gst-LckSH2. It also bound, to a lesser degree, to FynSH2, but not SrcSH2 or GST alone (Additional file 3). The functions of Odin in CRC are yet unknown and we have so far failed to see a direct complex formation of Odin and EphA8 in SW620 cells (data not shown). Immunoprecipitation of Odin with a specific antiserum and subsequent immunoblotting with anti-pY mAb showed that Odin is tyrosine phosphorylated. Treatment of SW620 cells with the SFK inhibitor PP2 [24,25], but not compound solvent alone, greatly reduced the detectable tyrosine phosphorylation of Odin, indicating that Odin is a SFK substrate, or a substrate of another tyrosine kinase that is regulated by SFK (Figure 5). SU6656, and Src-1, two other small molecule SFK inhibitors [25,26] were also tested, but found to be much less potent than PP2 in reducing overall pY levels in SW620 cells and did not effectively inhibit Odin tyrosine phosphorylation (not shown). We also tested the PP2-related inhibitor compound PP3 (4-amino-7-phenylpyrazol [3,4-d]pyrimidine) which does not inhibit SFK, but affects the EGF receptor [27] and did not observe a reduction of pY Odin (data not shown).

## Discussion

### Relatives and functions of Odin

Odin belongs into the small family of ANKS proteins that contains, apart from Odin (ANKS1A, KIAA0229, formerly a.k.a. ANKS; gene on chromosome 6p21.31), only one other member, ANKS1B (a.k.a. EB-1, AIDA-1 [AIDA-1a and AIDA-1b isoforms], cajalin-2, ANKS2; gene on chromosome 12q23.1). Odin and AIDA-1B are most closely related and share a similar structure. Odin contains multiple ankyrin repeats near its N-terminus as well as two SAM domains and, in the C-terminal half of the protein, a PTB (PID) domain, which belongs to the Dab-like subtype of PTBs [28]. These domains are connected by an intermediate region with no obvious structural elements, which harbours the majority of currently known phosphorylation sites. Two Odin pY sites have been repeatedly reported, namely pY454 and pY833 [29-32], but if and how they regulate Odin function, or which kinases are responsible for which site, remains unclear.

From its structure, Odin appears to be an adaptor protein, presumably coupling receptors like EGFR, PDGFR and EphA8 to their downstream signalling machinery. The roles of Odin in CRC, if any, remain to be elucidated. Knockdown of Odin in CRC cells with RNA interference may give further insight. However, RNA interference analyses of CRC cells using transient transfection with synthetic oligonucleotides are so far technically challenging and optimisation experiments are ongoing. Alternatively, transfection with shRNAs and selection of stable clones can be attempted, but this may lead to biased results, especially if Odin would be involved in regulating cell proliferation and/or survival, unless a system with tightly regulatable RNA interference is used.

It also remains to be investigated which receptors are coupled to Odin in CRC cells and whether these are linked to SFK. In a previous study we have documented a connection between the HGF receptor, c-Met, and SFK, namely an activation of c-Met by SFK. From our current analysis, we cannot exclude that Odin is an indirect target of SFK and, for example, directly phosphorylated by c-Met. Co-immunoprecipitations aiming to detect potential c-Met and Odin complexes and inhibition of c-Met kinase activity with specific compounds are obvious experiments to be included in follow-up studies.

### Odin as a potential biomarker for monitoring SFK inhibitor activities *in vivo*

Odin phosphorylation could also be explored for its usefulness as a clinical marker in monitoring the effectiveness of SFK inhibitors currently used in trials with CRC patients, if highly specific Odin phosphoepitope antibodies can be generated. This is, however, not a trivial matter since antibody cross-reactivity is a big problem for tissue stainings, especially when looking at protein modifications like phosphorylation. Approximately 520 kinases are encoded in the human genome and as much as one third of all cellular proteins are believed to contain phosphoresidues. Many of these kinase substrate proteins come in multiple splice variants, creating additional complexity. The average kinase has been estimated to have around different 20 substrate proteins with an even greater number of substrate epitopes. In many cases, the substrates of a specific kinase will have substantial amino acid similarity in the kinase-targeted epitopes, making it likely that polyclonal phosphoepitope antibodies generated against a specific site will cross-react at least to some degree with other substrate epitopes of the same kinase. Generation of mAbs against a phospho-epitope of interest can sometimes, albeit not always, reduce or eliminate cross-reactivity problems. Another approach to overcome cross-reactivity problems is the use of FRET [33] or similar methods, but these techniques are still under development and not yet part of the tool repertoire routinely used in clinical practice.

### Identification of additional tyrosine targets in epithelial cancer cells and beyond

Since cellular protein – protein interactions depending on pY-containing epitopes are predominantly mediated by SH2 domains, we have explored in this pilot study their usefulness for the rapid MS identification of novel tyrosine kinase targets in CRC cells. Approximately 120 SH2s, which differ considerably in their pY-epitope binding selectivity, have been recognised in the human genome to date [34,35]. A subset of PTB domains and the C2 domain of PKCdelta have also been reported to bind to specific pY-containing epitopes in cells [28,36]. Of the 120 exisiting human SH2 domains, over 80 can be expressed in bacteria and are also already functionally validated [34,37]. Together with a much smaller set of PTBs, they should become a valuable resource to quickly identify many novel substrate proteins in tyrosine kinase-driven cancers and many other diseases. SH2 domain-based affinity matrices may even prove to be more effective than current standard affinity purifications relying on anti-pY mAbs and they could potentially also preferentially enrich for proteins with biologically relevant modifications.

Beyond this, and taking into consideration the current pace of advances in MS techniques, even the identification of whole SH2 interactomes representing a systematic analysis of all binding partners for SH2 domains in a specific organism may be lurking just behind the horizon.

## Conclusion

SH2 domain-based affinity chromatography combined with MS analysis allows for the rapid identification of tyrosine kinase targets in human cancer cells, which should facilitate the elucidation of new signalling mechanisms, support the identification of new biomarker candidates and potentially even point to novel therapeutic targets. In the current study, the adaptor protein Odin was identified as a new SFK target in CRC cells that can now be analysed for a potential role in CRC development.

## Methods

### CRC lines, total cell lysates and cytosolic extracts

Cell line origins, culture conditions and total cell protein extract preparations of 64 CRC lines with RIPA buffer [20 mM TrisHCl pH 7.5, 100 mM NaCl, 1 mM EDTA, 1% Triton X-100, 0.5% deoxycholic acid, 0.1% SDS; supplemented with 2× Complete™ protease inhibitor mix (11697498001; Roche) and phosphatase inhibitor cocktails 1 and 2 (P2850 and P5726; Sigma)] were previously described [5]. SW480 cells (origin: ATCC) are derived from the same patient from which SW620 cells were made. SW480 were established from the primary tumor, SW620 from a lymph node metastasis [38]. To prepare the cytosolic extracts (S100) from SW620, cells were washed thrice with chilled PBS and once with hypotonic lysis buffer [HLB; 10 mM TrisHCl (pH 7.5), 10 mM KCl, 1 mM EDTA, 1 mM EGTA, 2 mM MgCl_2_, 1 mM DTT, supplemented with 2× Complete™ protease inhibitor mix and phosphatase inhibitor cocktails 1 and 2 (P2850 and P5726)] and then scraped and fully lysed by dounce homogenization in HLB. The homogenate was then clarified by high-speed centrifugation (1 h, 100,000 × g, 4°C) to obtain S100, which was snap-frozen with liquid nitrogen in aliquots and stored at -80°C until further use. Protein concentrations were determined by the Bradford method [39].

### Expression and purification of GST-SH2 fusion proteins

The production of GST and different GST-SH2 fusion proteins [GST-AblSH2, GST-CrkLSH2, GST-FynSH2, GST-GapSH2 (N-terminal), GST-GrapSH2, GST-LckSH2, GST-Mona(Gads)SH2, GST-NckSH2, GST-PI3KSH2 (C-terminal), GST-PLCgammaSH2 (N-terminal), GST-ShcSH2, GST-SrcSH2, GST-Shp2SH2 (C-terminal), GST-TensinSH2 and GST-Vav1SH2] was described previously [40-43]. Briefly, after affinity purification on GSH-sepharose, elution with free GSH and three-fold dialysis in 5 mM TrisHCl (pH 7.5) to remove GSH, protein concentrations were determined by Bradford assay and protein purity and integrity analysed by SDS-PAGE followed by staining with Coomassie Blue (Brilliant Blue R, B-0630; Sigma).

### Western blots and immunoprecipitations (IPs)

Western blotting of proteins was done as essentially previously described [5]. Anti-Lck (mAb 3A5) was from Santa Cruz Biotechnology (sc-433). pY proteins were detected with anti-pY mAb (4G10). 1 μg of anti-Odin (rabbit polyclonal, ST1039; Calbiochem) was used for IP using 1 mg of total cell RIPA protein extract incubating with 20 μl of protein A beads overnight at 4°C. Normal rabbit IgG (sc-2027) was used for control IPs. Lck IPs were done with 4 μg anti-Lck mAb for 1 mg of total cell RIPA protein extract. An equal amount of anti-FLAG mAb (M2; Kodak) was used as IgG isotype control. Both mAb were captured with protein G beads. Anti-pY419Src (polyclonal rabbit, 2101; Cell Signaling Technology) reacts with all members of the SFK family expressed in CRC cells due to high conservation of the target epitope (for further details see [5]). It was used to simultaneously analyse multiple family members in total cell lysates, or to specifically investigate Lck phosphorylation in the kinase activation loop after Lck IP. Cell extracts were also probed for actin (mAb, A3853; Sigma-Aldrich) as a loading control. Molecular weight standards used were from GE Healthcare (RPN756 and RPN800E).

### Treatment of SW620 cells with kinase inhibitors

SW620 cells were incubated with 30 μM SFK inhibitor PP2 (529573; Calbiochem) dissolved in DMSO, or the structural analogue PP3, which lacks SFK inhibitory activity (529574; Calbiochem), or with the compound solvent (D2650; Sigma-Alrich) alone for 24 h. Cells were then harvested by lysing in RIPA buffer [5] and protein concentrations determined by Bradford assay.

### Precipitation assay with GST-SH2 domains

GST-SH2 precipitation assays to analyse pY protein binding were done at 4°C with total cell RIPA lysates. 1 mg of protein extract was mixed in each case with GSH beads loaded with either 50 μg GST or GST-SH2 fusion protein. After nutating in the cold overnight, beads with captured proteins were washed 4 times with chilled RIPA buffer [5] and bound proteins separated by SDS-PAGE. After transfer to PVDF membrane, pY proteins were visualized by immunoblotting with anti-pY mAb.

### Enrichment of pY proteins for mass spectrometry analysis

S100 (5–20 mg per sample) was initially precleared with 100 μl of GSH beads pre-coupled with 200 μg of GST for 3 hours at 4°C, after which beads were spun down and supernatants were recovered. This procedure was then repeated once, after which the supernatant was pre-cleared once more with empty GSH beads to remove traces of GST, presumed to have leaked off previously. Precleared S100 was then incubated overnight with GSH beads loaded either with 100 μg GST-LckSH2 or 100 μg GST-LckSH2 pre-incubated overnight with 100 μg of EPQpYEEIPI peptide (amidated at C-terminus, HPLC-purified). The beads were then washed four times with chilled RIPA buffer and samples subjected to SDS-PAGE. After electrophoresis, the gels were stained with Coomassie Brilliant Blue R, or Roti-Blue Colloidal Coomassie staining (Carl Roth, Germany) according to manufacturer's instruction.

### Identification of SH2-interacting proteins by tandem mass spectrometry

The gel region containing proteins of ca. 45 kDa or more were excised cut into 10 separate slices representing distinct molecular weight regions and subjected to in-gel trypsin digestion as previously described [44]. Digested protein material was kept at 4°C until analysis. Sample analysis was performed by LC-MS/MS using an Ultimate™ (LC-Packings, Dionex, Amsterdam, NL) HPLC system coupled on-line to a 3D high-capacity ion trap (HCTplus™, Bruker Daltonics, Bremen, Germany) mass spectrometer via a pneumatically assisted nano-electrospray source as described previously [45]. MS/MS spectra (peak lists) were searched against the SwissProt (release 54.0, 07/2007, number of entries 276256) or trEMBL (release 37.0, 07/2007) databases using Mascot version 2.2 (Matrixscience, London, UK) and the following parameters: peptide tolerance 2.5 Da, ^13^C=0, fragment tolerance 0.8 Da, missed cleavages: 3, instrument type: ESI-TRAP. The interpretation and presentation of MS/MS data was performed according to published guidelines [46]. In addition, individual MS/MS spectra for peptides with a Mascot Mowse score lower than 40 (Expect <0.015) were inspected manually and included in the statistics only if a series of at least 4 continuous y or b ions were observed. Protein ID is also based on the assignment of at least two peptides. In cases where proteins were identified based on one peptide sequence, the corresponding MS/MS spectra were inspected and verified manually.

## Abbreviations

CRC: colorectal cancer; GSH: glutathione; GST: glutathione S-transferase; IP immunoprecipitation; mAb: monoclonal antibody; MS: mass spectrometry/mass spectrometric; pY: phosphotyrosine/phosphotyrosyl/phosphorylated tyrosine; SFK: Src family kinase(s); TCL: total cell lysate.

## Competing interests

The authors declare that they have no competing interests.

## Authors' contributions

ME designed and carried out experiments, analysed data and co-drafted the manuscript. MJE performed mass spectrometric experiments and analysed data. BK analysed data and critically commented on the drafted manuscript. SF conceived the project, contributed to experimental design and conduction of experiments, analysed data and drafted the manuscript.

## Supplementary Material

Additional File 1 **Comparison of SW480 and SW620 CRC cell morphologies.** Cells are grown on cell culture plastic without extra coating. Both images were taken ca. 60 h after cell passaging and are shown at the same magnification. The SW480 cells, which are derived from primary tumor tissue appear more attached. SW620 cells, derived from a lymph node metastasis of the same patient appear to be on average smaller and more spindle shaped. Similar morphological changes are, for example, also observed with fibroblasts upon transformation by SFK. Click here for file

Additional File 2 **Supplementary Table.** Click here for file

Additional File 3 **In vitro binding of Odin to the LckSH2 domain.** 1 mg of SW620 total cell RIPA lysate (TCL) was precipitated with 50 μg of GSH bead-immobilised GST or GST-SH2 fusion protein or GST-LckSH2 preincubated with a specific blocking pY-peptide and then washed three times with a 1% Triton X-100 containing buffer. Precipitated proteins were separated by SDS-PAGE and analysed by western blot with anti-Odin. 2 μg of TCL was loaded for comparison. Odin binding appears to be most prominent to the LckSH2 domain. The identity of the band prominently precipitated with the FynSH2 is unclear. It could be, for example, a splice variant, a proteolytic cleavage product of Odin or a cross-reactive other protein. Click here for file
