# Supplementary material for: Odin (ANKS1A) is a Src family kinase target in colorectal cancer cells
Source: Cell Commun Signal. 2008 Oct 9;6:7. doi: 10.1186/1478-811X-6-7 (PMC2584000; doi:10.1186/1478-811X-6-7)
Supplement: Additional File 1 — Comparison of SW480 and SW620 CRC cell morphologies. Cells are grown on cell culture plastic without extra coating. Both images were taken ca. 60 h after cell passaging and are shown at the same magnification. The SW480 cells, which are derived from primary tumor tissue appear more attached. SW620 cells, derived from a lymph node metastasis of the same patient appear to be on average smaller and more spindle shaped. Similar morphological changes are, for example, also observed with fibroblasts upon transformation by SFK. [file 1478-811X-6-7-S1.ppt]

## Slide 1
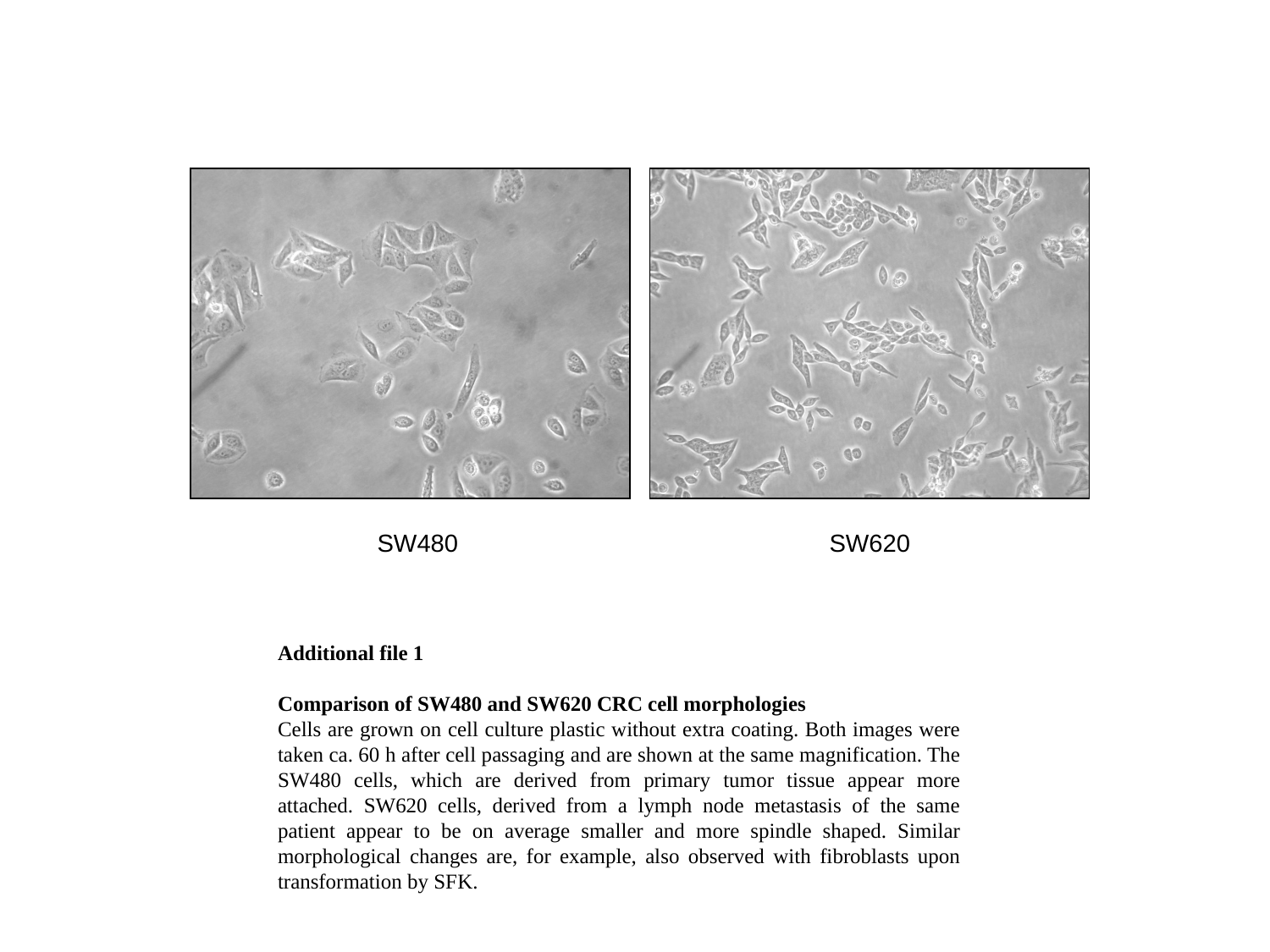

SW480
SW620
Additional file 1
Comparison of SW480 and SW620 CRC cell morphologies
Cells are grown on cell culture plastic without extra coating. Both images were taken ca. 60 h after cell passaging and are shown at the same magnification. The SW480 cells, which are derived from primary tumor tissue appear more attached. SW620 cells, derived from a lymph node metastasis of the same patient appear to be on average smaller and more spindle shaped. Similar morphological changes are, for example, also observed with fibroblasts upon transformation by SFK.
