# Supplementary material for: Odin (ANKS1A) is a Src family kinase target in colorectal cancer cells
Source: Cell Commun Signal. 2008 Oct 9;6:7. doi: 10.1186/1478-811X-6-7 (PMC2584000; doi:10.1186/1478-811X-6-7)
Supplement: Additional File 3 — In vitro binding of Odin to the LckSH2 domain. 1 mg of SW620 total cell RIPA lysate (TCL) was precipitated with 50 μg of GSH bead-immobilised GST or GST-SH2 fusion protein or GST-LckSH2 preincubated with a specific blocking pY-peptide and then washed three times with a 1% Triton X-100 containing buffer. Precipitated proteins were separated by SDS-PAGE and analysed by western blot with anti-Odin. 2 μg of TCL was loaded for comparison. Odin binding appears to be most prominent to the LckSH2 domain. The identity of the band prominently precipitated with the FynSH2 is unclear. It could be, for example, a splice variant, a proteolytic cleavage product of Odin or a cross-reactive other protein. [file 1478-811X-6-7-S3.ppt]

## Slide 1
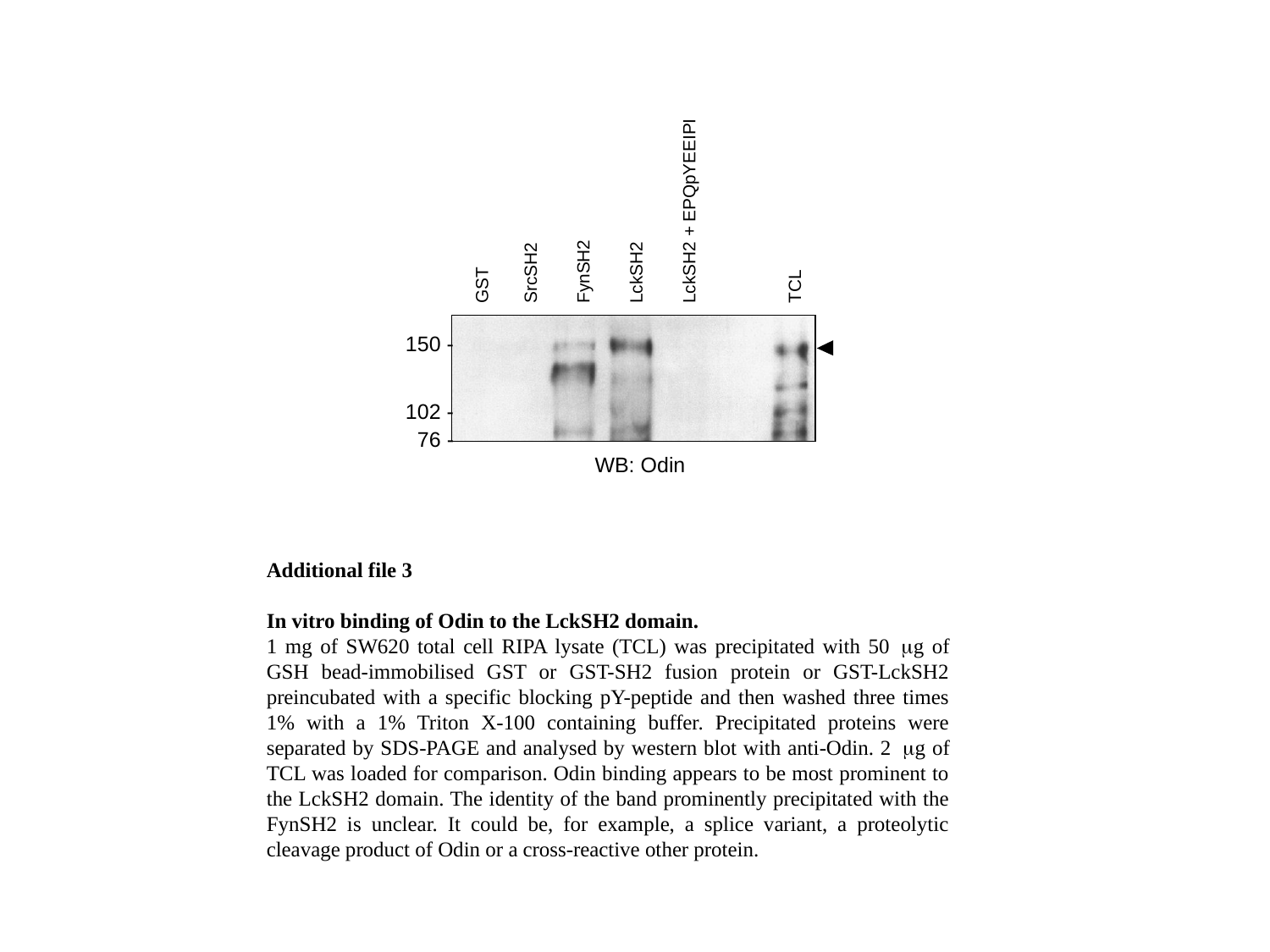

GST
SrcSH2
FynSH2
LckSH2
LckSH2 + EPQpYEEIPI
TCL
150 -
102 -
76 -
WB: Odin
Additional file 3
In vitro binding of Odin to the LckSH2 domain.
1 mg of SW620 total cell RIPA lysate (TCL) was precipitated with 50 μg of GSH bead-immobilised GST or GST-SH2 fusion protein or GST-LckSH2 preincubated with a specific blocking pY-peptide and then washed three times 1% with a 1% Triton X-100 containing buffer. Precipitated proteins were separated by SDS-PAGE and analysed by western blot with anti-Odin. 2 μg of TCL was loaded for comparison. Odin binding appears to be most prominent to the LckSH2 domain. The identity of the band prominently precipitated with the FynSH2 is unclear. It could be, for example, a splice variant, a proteolytic cleavage product of Odin or a cross-reactive other protein.
